# Nutrition assessment and MASH severity in children using the Healthy Eating Index

**DOI:** 10.1097/HC9.0000000000000320

**Published:** 2023-12-07

**Authors:** Ajay Kumar Jain, Paula Buchannan, Katherine P. Yates, Patricia Belt, Jeffrey B. Schwimmer, Philip Rosenthal, Karen F. Murray, Jean P. Molleston, Ann Scheimann, Stavra A. Xanthakos, Cynthia A. Behling, Paula Hertel, Jamie Nilson, Brent A. Neuschwander-Tetri, James Tonascia, Miriam B. Vos

**Affiliations:** 1 Department of Pediatrics, Division of Pediatric Gastroenterology, Hepatology, and Nutrition, Saint Louis University, St. Louis, Missouri, USA; 2 Department of Epidemiology, Johns Hopkins Bloomberg School of Public Health, Baltimore, Maryland, USA; 3 Department of Pediatrics, Division of Gastroenterology, UC San Diego, La Jolla, California, USA; 4 Department of Gastroenterology, Rady Children’s Hospital, San Diego, California, USA; 5 Department of Pediatrics, Division of Gastroenterology, Hepatology, and Nutrition, University of California, San Francisco Benioff Children’s Hospital, San Francisco, California, USA; 6 Pediatrics Institute, Cleveland Clinic and Cleveland Clinic Children’s Hospital, Cleveland, Ohio; 7 Department of Pediatrics, Division of Pediatric Gastroenterology, Hepatology and Nutrition, Department of Pediatrics, Indiana University School of Medicine/Riley Hospital for Children, Indianapolis, Indiana, USA; 8 Department of Pediatrics, Division of Pediatric Gastroenterology, Hepatology, and Nutrition, Johns Hopkins University, Baltimore, Maryland, USA; 9 Steatohepatitis Center, Division of Pediatric Gastroenterology, Hepatology and Nutrition, Cincinnati Children’s Hospital Medical Center, Cincinnati, Ohio, USA; 10 Department of Pediatrics, Division of Gastroenterology, UC San Diego, La Jolla, California, USA; 11 Department of Gastroenterology and Pacific Rim Pathology, San Diego, California, USA; 12 Division of Gastroenterology, Hepatology and Nutrition, Department of Pediatrics, Texas Children’s Hospital, Baylor College of Medicine, Houston, Texas, USA; 13 Division of Gastroenterology and Hepatology, Saint Louis University, St. Louis, Missouri, USA; 14 Department of Pediatrics, Division of Gastroenterology, Hepatology and Nutrition, Emory University School of Medicine, Children’s Healthcare of Atlanta, Atlanta, Georgia, USA

## Abstract

**Background:**

Pediatric metabolic-associated fatty liver disease (MAFLD) is a global health problem, with lifestyle modification as its major therapeutic strategy. Rigorous characterization of dietary content on MAFLD in children is lacking. We hypothesized an objectively measured healthier diet would positively modulate MAFLD.

**Methods:**

Diet was assessed using the Nutrition Data System for Research in children enrolled from 10 tertiary clinical centers to determine the Healthy Eating Index (HEI, 0–100) and individual food components.

**Results:**

In all, 119 children were included (13.3 ± 2.7 y), 80 (67%) male, 67 (18%) White, and 90 (76%) Hispanic, with an average body mass index Z-score of 2.2 ± 0.5. Diet was classified as low HEI < 47.94 (n = 39), mid HEI ≥ 47.94 and < 58.89 (n = 41), or high HEI ≥ 58.89 (n=39). Children with high HEI (healthier diet) had lower body weight (*p* = 0.005) and more favorable lipids. Mean serum triglycerides for low, mid, and high HEI were 163, 148, and 120 mg/dL, respectively; *p* = 0.04 mid versus high, *p* = 0.01 low versus high. Mean HDL was 38, 41 and 43 mg/dL; *p* = 0.02 low vs high. Less severe steatosis was noted with added sugar ≤ 10% of calories (*p* = 0.03). Higher lobular inflammation is associated with a higher percentage of calories from fat (OR (95% CI) = 0.95 (0.91–1.00), *p* = 0.04).

**Conclusions:**

In children with MAFLD, high HEI is associated with lower body weight and more favorable lipids, while added sugar and fat intake has individual histologic features. Differential consumption of major dietary components may modify both metabolic risk factors and histologic liver injury, highlighting the importance of objective diet assessments in children with MAFLD.

## INTRODUCTION

Metabolic-associated steatohepatitis (MASH) remains a major health problem worldwide.^1–3^ Though several therapeutic interventions have been trialed, the mainstay of current therapy remains lifestyle modification inclusive of improved nutrition.^1,4^ A “healthy” diet specifically for metabolic-associated fatty liver disease (MAFLD) is not well defined. It is likely to consist of increased micronutrient sources, sufficient protein levels, lower simple sugars, and calories to match energy needs.^5–7^

An objective way to assess diet quality is the Healthy Eating Index (HEI).^8^ The HEI uses a standardized scoring system to evaluate any set of foods recorded using 24-hour dietary recalls. The overall HEI score is made up of 13 components that reflect the different food groups and key recommendations and is scored from 0 to 100. An ideal overall HEI score of 100 reflects that the set of foods aligns with key dietary recommendations from the Dietary Guidelines for Americans.^9,10^

Several studies have reported an association of MASH with a high fructose diet^11,12^; however, large multicenter descriptions of diet in children with MAFLD are lacking, particularly using a detailed database of nutrient and caloric content of foods such as the Nutrition Data System for Research (NDSR).^8^ Recent literature also suggests associations between protein^13,14^ and fat^15–17^ intake and liver histology, but this has not been evaluated in pediatric populations with MAFLD.

Utilizing a well-characterized cohort of pediatric patients with biopsy-proven MAFLD who participated in the National Institute of Diabetes and Digestive and Kidney Diseases-supported nonalcoholic steatohepatitis Clinical Research Network (NASH CRN), “Cysteamine bitartrate delayed-release (CBDR) for the treatment of MAFLD in Children (CyNCh)”, randomized clinical trial (RCT) (NCT01529268)^18^ and NDSR, we hypothesized that higher added sugar intake is associated with greater hepatic injury, inflammation, and fibrosis and that longitudinally histology will worsen in those with an added sugar intake > 10% of calories compared to those with less added sugar intake. We also hypothesized that children with a higher protein intake will have a lower severity of hepatic steatosis and inflammation and that a diet with a higher HEI score will be associated with lower severity of hepatic injury and lower odds of progression of fibrosis over time in children with MAFLD.

## METHODS

**FIGURE 1:**
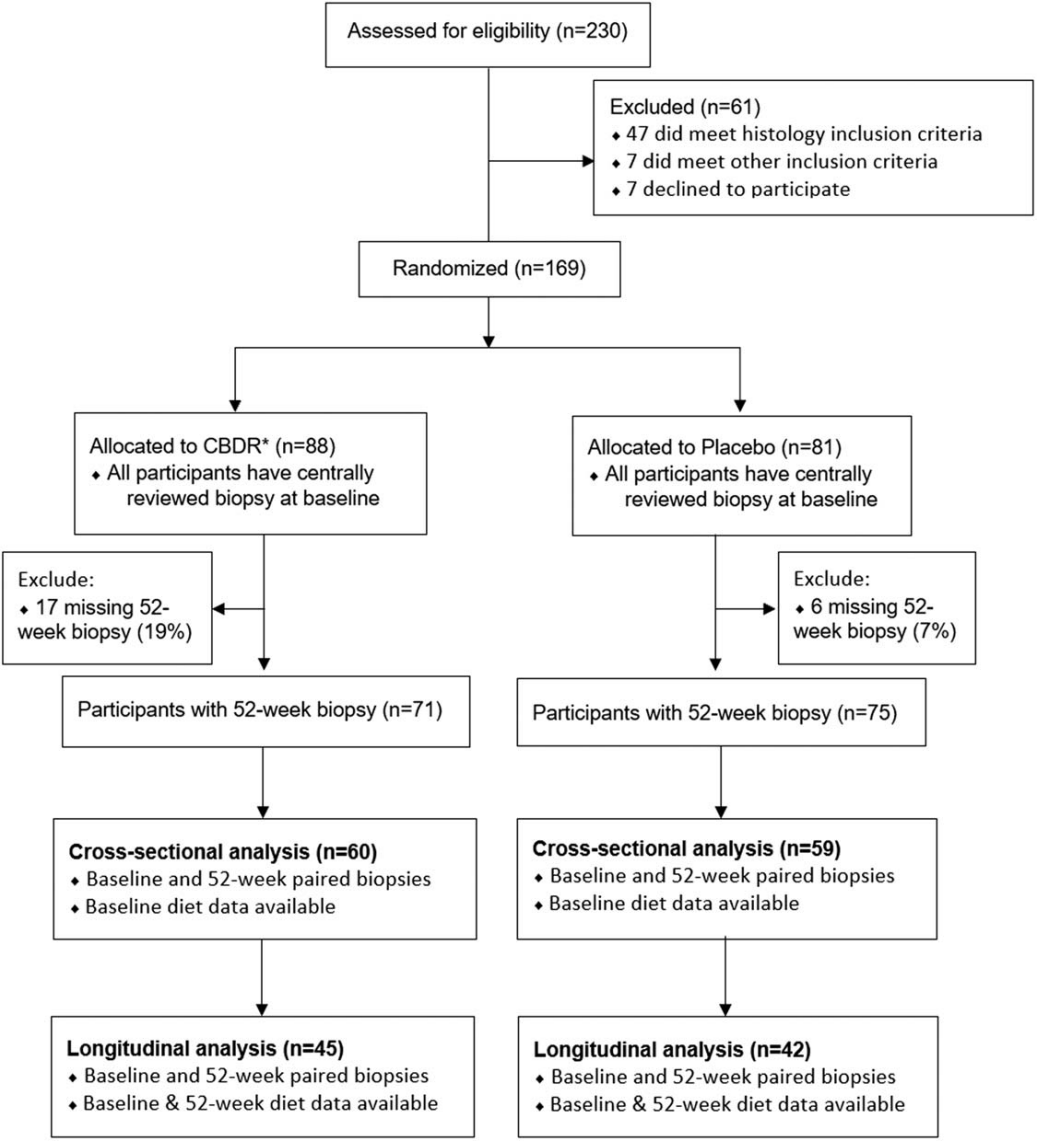
Shown here is the CONSORT diagram. Abbreviation: CBDR, Cysteamine bitartrate delayed-release.

Dietary food analysis data captured during the CyNCh RCT, enrolling pediatric patients 8–17 years old with biopsy-proven MAFLD^18^ from 10 US tertiary sites from June 2012 through January 2014 were utilized.^6^ The RCT methods and primary results have been published^18^ (Supplemental Appendices 1,2, http://links.lww.com/HC9/A690). Detailed diet records were obtained for each patient and analyzed using the NDSR. Up to 3 nonconsecutive days (including 1 weekend day) of dietician-administered 24-hour daily food intake recalls were collected separately at baseline and at 52 weeks. Subjects were excluded from this analysis if they had no intake diaries at baseline or did not have both baseline and 52-week histology. Within the CyNCh RCT, all children received a standardized nutrition and exercise intervention consistent with the American Academy of Pediatrics Expert Committee Recommendations Regarding the Prevention, Assessment, and Treatment of Child and Adolescent Overweight and Obesity and were treated with either placebo or cysteamine bitartrate. As recommended for MAFLD, lifestyle advice was provided at each study visit. A CONSORT diagram is included in Figure 1.

All research was conducted in accordance with both the Declarations of Helsinki and Istanbul. All research was approved by the institutional review committees at NASH CRN. All participants in the study provided written assent to the study, and parents or guardians provided written consent for their children to participate.

### Liver histologic features

At the beginning and end of the study, liver histology and liver chemistries were assessed. Liver pathology was scored centrally using a consensus review by the NASH CRN Pathology Committee. Examined liver histologic features included nonalcoholic fatty liver disease activity score (NAS), the individual components of NAS: lobular inflammation, steatosis, and ballooning, as well as fibrosis at baseline and 52 weeks.

NAS was assessed on a scale of 0 to 8. The components of NAS were the grade of steatosis (0–3), lobular inflammation (0–3), and hepatocellular ballooning (0–2). Other histologic features included fibrosis stage (0–4), portal inflammation grade (0–2), and steatohepatitis diagnosis (no MAFLD; MAFLD, but not steatohepatitis; borderline steatohepatitis with zone 3 pattern, borderline steatohepatitis with zone 1 pattern; or definite steatohepatitis). Higher scores were indicative of more severe inflammation or fibrosis.

For this analysis, histologic improvement was defined as a decrease in NAS of 2 or more points and no worsening of fibrosis. In addition, resolution of MASH was defined as a diagnosis of definite NASH at baseline and a diagnosis of not MAFLD or MAFLD only but not MASH at the 52-week follow-up.

### Nutritional intake

Overall nutritional intake was measured using The HEI 2015.^8^ The HEI ranges from 0 to 100, with a higher score reflecting that the set of foods aligns with key dietary recommendations from the Dietary Guidelines for Americans. The food recall diaries collected at the baseline and follow-up visits were utilized to calculate the daily HEI score. The baseline and follow-up HEI scores were computed as the average of the daily scores at each time point. HEI was categorized into low (< 25th percentile), mid (25th–75th percentile), and high (> 75th percentile) to indicate diet quality. In addition, the percent of calories from added sugar, protein, fat, and carbohydrates were calculated at baseline and follow-up. The percent of calories from sugar intake was categorized as > and ≤ 10% of diet, and the percent from added protein was categorized as > and ≤ 20%. Changes in the nutritional intake scores were calculated as the score at 52 weeks minus baseline.

### Subject demographic and clinical characteristics

Additional demographic and clinical characteristics collected included participant age (y) categorized as < 13 years versus ≥ 13 years as a proxy for onset of puberty; sex; race/ethnicity; weight (kg); height (m); body mass index (BMI)-z-score; sex; hemoglobin A1c; aspartate aminotransferase, U/L); alanine aminotransferase, U/L); gamma-glutamyl transferase, U/L); triglycerides (mg/dL); total cholesterol (mg/dL); HDL and LDL cholesterol (mg/dL); fasting serum glucose (mg/dL); fasting serum insulin (µU/mL); and medications.

### Statistical analysis

Diet quality groups were compared pairwise using chi-square tests for categorical characteristics. Associations with continuous characteristics were tested with *t*-tests for means and Mann-Whitney *U* tests for medians if the distribution was skewed. Bivariate associations with baseline HEI scores were computed through unadjusted linear regressions.

Baseline relationships between HEI and liver histologic features were examined using unadjusted binary logistic regressions for dichotomous features and ordinal logistic regressions modeling the probability of higher or more severe outcomes for the ordinal outcomes, with the independent variable being a 10 U difference in HEI scores. The test of proportionality was assessed for the ordinal logistic regressions and determined to be appropriate.

Improvement in liver histologic features defined as histologic improvement, resolution of MASH, or a one or more-point decrease in NAS, fibrosis, ballooning, lobular inflammation, portal inflammation, and steatosis at 52 weeks from baseline were examined. The relationship between 10 U change in HEI score and improvements in the histologic features was assessed with binary logistic regressions and receiver operating characteristics analysis. The regressions and area under the receiver operating characteristic curve (AUROC) analysis were adjusted for CyNCh treatment group, age < 13 versus ≥13, baseline BMI, baseline HEI score and baseline outcome. ORs and AUROCs were calculated along with their 95% CIs.

Finally, to assess the association between a 1-year U change in histology score for each additional 10 U increase of HEI score were computed from bootstrapped linear regression models based on 1000 samples. Both unadjusted and adjusted models were run on NAS, fibrosis, ballooning, lobular inflammation, portal inflammation, and steatosis. Models were adjusted for the CyNCh treatment group, age < 13 versus ≥13, baseline BMI z-score, and baseline HEI and histology scores.

The effects of added sugar, % calories from protein, % calories from fat, and % calories from carbohydrates were examined against the outcomes of liver histologic features, with added sugar being categorized as > and ≤ 10% and protein as > and ≤ 20% total calories. Improvement and changes in histologic features were compared to the HEI score, utilizing a 1% change in the nutrition component and adjusting the analysis for the CyNCh treatment group, age < 13 vs ≥13, baseline BMI, baseline nutrition component, and baseline outcome.

One-year change in HEI and dietary components were assessed with paired *t*-test. Baseline dietary components were compared between CyNCh treatment groups with independent samples *t*-tests.

All analyses were done in SAS v9.4 at a significant level of 0.05. No adjustments were made for multiple comparisons.

## RESULTS

### Baseline characteristics

There were 119 children with diet data at baseline (mean age 13.3 ± 2.7 y, 80 (67% male) and 90 (75%) Hispanic) in this study, of which 87 also had follow-up histology and diet data over a year. Of the 119 at baseline, 116 (97.5%) had 3 days of food diaries and 81 (93%) had 3 days of food diaries at follow-up. Comparing the HEI groups at baseline (Table 1), those with a lower HEI score were older than those with the healthiest HEI scores (*p* = 0.005). There were no differences in age for those with mid HEI versus low and mid versus high.

**TABLE 1:** Baseline subject characteristics

	Total (N = 119)	Low HEI^a^ (n = 30)	Mid HEI^a^ (n = 59)	High HEI^a^ (n = 30)	Low vs. mid HEI *p*-value^b^	Mid vs. high HEI *p*-value^b^	Low vs. high HEI *p*-value^b^	β^c^ (95% CI)	*p*
Age (y), mean (SD)	13.3 (2.67)	14.1 (2.2)	13.4 (2.9)	12.4 (2.5)	0.10	0.054	0.005	−1.12 (−1.94 to −0.31)	0.007
< 13, n (%)	59 (49.6)	9 (30.0)	30 (50.8)	20 (66.7)	0.06	0.16	0.004	Reference	
BMI z-score, mean (SD)	2.19 (0.45)	2.29 (0.45)	2.20 (0.39)	2.08 (0.54)	0.18	0.27	0.11	−6.70 (−11.5 to −1.89)	0.007
Weight (kg), median (IQR)	84.2 (60.2 to 99.4)	95.2 (66.3 to 112.1)	81.9 (63.2 to 96.5)	62.7 (51.8 to 94.4)	0.04	0.10	0.005	−0.15 (−0.23 to −0.07)	<0.001
Biopsy length (mm), median (IQR)	18 (14 to 25)	16 (12 to 20.8)	18 (14 to 30)	20.5 (17.8 to 23.3)	0.10	0.40	0.03	0.08 (−0.12 to 0.28)	0.44
Hemoglobin (g/dL), median (IQR)	13.6 (13.1 to 14.5)	13.7 (13.0 to 14.6)	13.8 (13.2 to 14.7)	13.2 (12.8 to 13.8)	0.66	0.01	0.13	−1.81 (−3.68 to 0.07)	0.06
White blood cell count (WBC) median (IQR)	7.4 (6.5 to 9.2)	8 (6.5 to 9.8)	7.4 (6.5 to 8.8)	7.1 (6.0 to 8.5)	0.44	0.50	0.21	−1.17 (−2.21 to −0.13)	0.03
Platelets (cells/mm^3), mean (SD)	291134 (66490)	295033 (73829)	286864 (64742)	295633 (63880)	0.30	0.55	0.97	0.00 (0.00 to 0.00)	1.00
HbA1c (%), median (IQR)	5.5 (5.2 to 5.7)	5.3 (5.1 to 5.6)	5.5 (5.2 to 5.7)	5.5 (5.2 to 5.6)	0.10	0.74	0.25	1.70 (−3.27 to 6.66)	0.50
AST (U/L), median (IQR)	51 (38 to 86)	42.5 (32.5 to 88.8)	54.0 (40 to 81)	49 (41.3 to 78.5)	0.28	0.93	0.29	−0.02 (−0.05 to 0.02)	0.31
ALT (U/L), median (IQR)	84 (60 to 157)	77 (60 to 147.5)	96 (53 to 160)	86 (64 to 147)	0.77	0.74	0.86	−0.01 (−0.03 to 0.01)	0.33
GGT (U/L), median (IQR)	37 (27 to 67)	37.5 (29.3 to 73.8)	38 (27 – 67)	33 (27 to 51.5)	0.64	0.30	0.20	−0.06 (−0.13 to 0.001)	0.05
Triglycerides (mg/dl) median (IQR)	146 (98 to 202)	162.5 (108.5 to 229.5)	148 (98 to 210.3)	120 (68.5 to 154.8)	0.38	0.04	0.01	−0.04 (−0.06 to −0.01)	0.01
Total cholesterol (mg/dL), mean (SD)	165.91 (37.94)	165.5 (40.1)	172.0 (37.4)	154.5 (35.2)	0.23	0.04	0.26	−0.05 (−0.11 to 0.01)	0.10
HDL cholesterol (mg/dL), mean (SD)	40.78 (9.55)	37.6 (6.8)	41.2 (10.3)	43.1 (9.9)	0.03	0.41	0.02	0.20 (−0.03 to 0.44)	0.09
LDL cholesterol (mg/dL), mean (SD)	94.79 (30.54)	94.8 (32.4)	99.0 (30.5)	86.9 (28.1)	0.55	0.07	0.32	−0.06 (−0.13 to 0.02)	0.14
Fasting serum glucose (mg/dL), median (IQR)	85 (79 to 90)	85 (78.5 to 90.5)	86 (80 – 93)	84 (71 – 89.3)	0.57	0.08	0.31	−0.13 (−0.31 to 0.04)	0.14
Fasting serum insulin (uU/mL), median (IQR)	28.1 (18 to 42.8)	31.1 (20.1 to 53.2)	28.7 (18 to 44)	29.5 (13.8 to 36)	0.44	0.55	0.28	−0.04 (−0.11 to 0.03)	0.29
Total calories (kcal), mean (SD)	1471.78 (430.01)	1568.8 (472.0)	1512.1 (391.5)	1295.4 (421.7)	0.27	0.02	0.02	−0.01 (−0.01 to −0.002)	0.01
Calories from fat (%), mean (SD)	31.17 (7.4)	35.7 (6.7)	32.0 (6.2)	24.9 (6.2)	0.005	<0.001	<0.001	−1.00 (−1.24 to −0.76)	<0.001
Calories from carbohydrates (%), mean (SD)	49.84 (7.36)	47.6 (7.0)	48.7 (6.3)	54.3 (7.9)	0.23	<0.001	<0.001	0.72 (−0.45 to 1.00)	<0.001
Calories from protein (%), mean (SD)	18.98 (4.85)	16.6 (4.1)	19.3 (4.1)	20.7 (6.0)	0.003	0.23	0.003	0.67 (0.22 to 1.12)	0.004
% calories added sugars (%), mean (SD)	9.5 (5.33)	13.0 (5.5)	9.3 (4.9)	6.4 (3.9)	<0.001	0.007	<0.001	−1.06 (−1.44 to −0.69)	<0.001
Added sugar > 10%	51 (42.9)	22 (73.3)	25 (42.4)	4 (13.3)	0.006	0.006	<0.001	−11.26 (−15.29 to −7.24)	<0.001
Added protein > 20%	45 (37.8)	6 (20.0)	27 (45.8)	12 (40.0)	0.02	0.60	0.09	3.55 (−1.02 to 8.12)	0.13

a Low: HEI < 43.93 (25th percentile), Mid: HEI ≥ 43.93 and HEI < 61.92 (25th–75th percentile), High: HEI ≥ 61.92 (75th percentile).

b Independent samples *t*-tests for comparing means, Mann-Whitney *U* test for comparing medians, and chi-square test for comparing percentages.

c β coefficient of simple linear regression on baseline HEI score.

Abbreviations: ALT, alanine aminotransferase; AST, aspartate aminotransferase; BMI, body mass index; CBDR, cysteamine bitartrate delayed-release; CyNCh, Cysteamine bitartrate delayed-release for the treatment of NAFLD in children; GGT, gamma-glutamyl transferase; HEI, Healthy Eating Index.

High HEI was associated with a significantly lower weight (*p* = 0.005). There was a significant negative relationship between BMI z-score and HEI score as a continuous variable [β (95% CI), −6.7 (−11.5 to -1.9, *p* = 0.007)]. No difference in BMI z-score by the categorical HEI or differences in HbA1c, aspartate aminotransferase, alanine aminotransferase, gamma-glutamyl transferase, fasting serum glucose, and insulin were noted. For the lipid components, triglycerides were the lowest in the high HEI group (*p* = 0.04 mid vs. high, *p* = 0.01 for low vs. high). HDL cholesterol was lower in the low HEI group compared to mid HEI (*p* = 0.03) and also in the low HEI group compared to high HEI (*p* = 0.02).

### Healthy Eating Index (HEI)

HEI was positively associated with lobular inflammation but no other liver histologic feature at baseline. While there were no significant differences between low versus mid HEI or mid versus high HEI, those with a low HEI score had less lobular inflammation compared to those with a high HEI score [mean (SD) 1.5 (0.6) vs 1.9 (0.7), *p* = 0.02, Table 2]. After 52 weeks, a 10-unit increase of HEI was associated with a higher lobular inflammation score (OR [95% CI] = 1.33 [1.002 to 1.77], *p* = 0.049, Table 3). While there were no associations with a 10 U change in HEI and improvement versus no improvement in liver histologic features after adjusting for the treatment group, age group, baseline BMI, baseline HEI score, and baseline outcome score, the modelling did predict most outcomes moderately better than chance alone based off the AUROC and corresponding 95% CI for histologic improvement, resolution of MASH, and a 1 point or greater improvement in NAS, fibrosis, lobular and portal inflammation, and steatosis. Of these, the model is the best at predicting an improvement in ballooning, AUC (95% CI) = 0.94 (0.90 to 0.99) In addition, there was a small negative association between 10 U change in HEI and change in lobular inflammation score after adjusting for covariates [B(95% CI) = −0.18 (−0.30 to −0.07), *p* = 0.007] (Table 4, Supplemental Table S-1, http://links.lww.com/HC9/A690). Longitudinally, there was poor correlation between change in the NAS score and the baseline HEI score (Supplemental Figure S1A, http://links.lww.com/HC9/A690) as well as a change in HEI with the baseline NAS (Supplemental Figure S1B, http://links.lww.com/HC9/A690).

**TABLE 2:** Baseline relationship between HEI and liver histologic features

	Total (N = 119)	Low HEI^a^ (n = 30)	Mid HEI^a^ (n = 59)	High HEI^a^ (n = 30)	Low vs. mid HEI *p*-value^b^	Mid vs. high HEI *p*-value^b^	Low vs. high HEI *p*-value^b^
NAS^c^, mean (SD)	4.66 (1.39)	4.4 (1.5)	4.9 (1.4)	4.6 (1.2)	0.07	0.42	0.25
NAS >= 5, n(%)	67 (56.3)	15 (50.0)	34 (57.6)	18 (60.0)	—	—	—
Steatohepatitis diagnosis^c^, n(%)	—	—	—	—	0.24	0.31	0.27
MAFLD	26 (21.8)	8 (26.7)	12 (20.3)	6 (20.0)	—	—	—
1a-borderline Zone 3	16 (13.4)	7 (23.3)	6 (10.2)	3 (10.0)	—	—	—
1b-borderline Zone 1	46 (38.7)	9 (30.0)	21 (35.6)	16 (53.3)	—	—	—
Definite	31 (26.1)	6 (20.0)	20 (33.9)	5 (16.7)	—	—	—
Fibrosis stage^c^, mean (SD)	1.30 (1.05)	1.37 (1.16)	1.36 (1.08)	1.13 (0.90)	0.48	0.15	0.19
Fibrosis stage^c^, n(%)							
0	29 (24.4)	8 (26.7)	14 (23.7)	7 (23.3)	0.88	0.53	NA
1	49 (41.2)	11 (36.7)	23 (39.0)	15 (50.0)	—	—	—
2	17 (14.3)	3 (10.0)	9 (15.3)	5 (16.7)	—	—	—
3 or 4	24 (20.1)	8 (26.6)	13 (22.0)	3 (10.0)	—	—	—
Ballooning, mean (SD)	0.61 (0.76)	0.53 (0.68)	0.71 (0.83)	0.47 (0.68)	0.16	0.07	0.35
Ballooning^c^, n(%)							
None	67 (56.3)	17 (56.7)	31 (52.5)	19 (63.3)	0.26	0.30	NA
Few	32 (26.9)	10 (33.3)	14 (23.7)	8 (26.7)	—	—	—
Many	20 (16.8)	3 (10.0)	14 (23.7)	3 (10.0)	—	—	—
Lobular inflammation^c^, mean (SD)	1.73 (0.71)	1.50 (0.63)	1.76 (0.75)	1.90 (0.66)	0.052	0.40	0.02
Lobular inflammation							
1	50 (42)	17 (56.7)	25 (42.4)	8 (26.7)	0.24	0.25	NA
2	51 (42.9)	11 (36.7)	23 (39.0)	17 (56.7)	—	—	—
3	18 (15.1)	2 (6.7)	11 (18.6)	5 (16.7)	—	—	—
Portal inflammation^c^, mean (SD)	1.13 (0.51)	1.13 (0.43)	1.14 (0.57)	1.14 (0.57)	0.49	0.77	0.78
Portal inflammation, n(%)							
None	9 (7.6)	1 (3.3)	6 (10.2)	2 (6.7)	NA	0.59	NA
Mild	86 (72.3)	24 (80.0)	39 (66.1)	23 (76.7)	—	—	—
More than mild	24 (20.2)	5 (16.7)	14 (23.7)	5 (16.7)	—	—	—
Steatosis grade^c^, n(%)							
1	22 (18.5)	5 (16.7)	10 (16.9)	7 (23.3)	0.90	0.72	0.81
2	36 (30.3)	10 (33.3)	17 (28.8)	9 (30.0)	—	—	—
3	61 (51.3)	15 (50.0)	32 (54.2)	14 (46.7)	—	—	—

a Low: HEI < 43.93 (25th percentile), Mid: HEI ≥ 43.93 and HEI < 61.92 (25th–75th percentile), High: HEI ≥ 61.92 (75th percentile).

b Independent samples *t*-tests for comparing means, Mann-Whitney *U* test for comparing medians, and chi-square test for comparing percentages. NA indicates the test could not be conducted due to the low number of subjects.

c NAS was assessed on a scale of 0-8, with higher scores showing more severe disease [the components of this measure are steatosis (assessed on a scale of 0-3), lobular inflammation (assessed on a scale of 0-3), and hepatocellular ballooning (assessed on a scale of 0-2)]. The fibrosis stage was assessed on a scale of 0-4 (by collapsing 1a,1b, and 1c to 1), with higher scores showing more severe fibrosis.

Abbreviations: HEI, Healthy Eating Index; MAFLD, metabolic-associated fatty liver disease; NAS, nonalcoholic fatty liver disease activity score.

**TABLE 3:** Liver histologic features^a^ and relationship with HEI score (per 10 units) at baseline

	Total (N = 119) n(%)	Baseline HEI score Mean (SD)	OR (95% CI)/10 HEI units^b^	*p*
NAS ≥ 5	67 (56.3)	54.0 (12.3)	1.06 (0.78–1.42)	0.72
Steatohepatitis diagnosis	—	—	0.97 (0.74 – 1.27)	0.83
MAFLD	26 (21.8)	53.7 (12.5)	—	—
1a-borderline zone 3	16 (13.4)	50.5 (12.3)	—	—
1b-borderline zone 1	46 (38.7)	56.2 (12.7)	—	—
Definite	31 (26.1)	51.6 (11.1)	—	—
Fibrosis stage	—	—	0.87 (0.66 – 1.14)	0.30
0	29 (24.4)	53.8 (12.5)	—	—
1	49 (41.2)	55.3 (12.7)	—	—
2	17 (14.3)	53.7 (11.1)	—	—
3 or 4	24 (20.1)	50.3 (11.9)	—	—
Ballooning	—	—	0.85 (0.63 – 1.13)	0.26
None	67 (56.3)	54.9 (13.0)	—	—
Few	32 (26.9)	51.7 (12.2)	—	—
Many	20 (16.8)	52.7 (9.7)	—	—
Steatosis grade	—	—	0.90 (0.68 – 1.19)	0.47
1	22 (18.5)	54.6 (12.3)	—	—
2	36 (30.3)	54.6 (13.1)	—	—
3	61 (51.3)	52.8 (11.9)	—	—
Lobular inflammation	—	—	1.33 (1.002 – 1.77)	0.049
1	50 (42)	51.0 (11.7)	—	—
2	51 (42.9)	55.3 (13.7)	—	—
3	18 (15.1)	56.6 (11.9)	—	—
Portal inflammation	—	—	0.92 (0.66 – 1.27)	0.60
None	9 (7.6)	58.6 (11.0)	—	—
Mild	86 (72.3)	53.1 (12.3)	—	—
More than mild	24 (20.2)	53.8 (12.8)	—	—

a NAS was assessed on a scale of 0-8, with higher scores showing more severe disease [the components of this measure are steatosis (assessed on a scale of 0-3), lobular inflammation (assessed on a scale of 0-3), and hepatocellular ballooning (assessed on a scale of 0-2)]. Fibrosis stage was assessed on a scale of 0-4 (by collapsing 1a,1b, and 1c to 1), with higher scores showing more severe fibrosis.

b Logistic regression for 2 category outcomes and ordinal logistic regression for ordinal outcomes. Test of proportionality was assessed for ordinal logistic regression, with the probability of higher/more severe outcome modeled.

Abbreviations: HEI, Healthy Eating Index; MAFLD, metabolic-associated fatty liver disease; NAS, nonalcoholic fatty liver disease activity score.

**TABLE 4:** Association between changes in HEI (10-unit change) and liver histologic features over time

	OR_ADJ_ (95% CI)/10-unit change in HEI^a^	*p*	AUROC^b^ (95% CI)
Histologic improvement^c^	1.26 (0.81 – 1.95)	0.06	0.66 (0.54–0.78)
Resolution of MASH	1.08 (0.63 – 1.84)	0.54	0.67 (0.51–0.82)
≥ 1point improvement			
NAS	1.54 (0.73–1.83)	0.54	0.78 (0.68–0.88)
Fibrosis	1.08 (0.67–1.76)	0.11	0.80 (0.71–0.89)
Ballooning	1.27 (0.62–2.58)	0.52	0.94 (0.90–0.99)
Lobular Inflammation	1.49 (0.87–2.56)	0.15	0.89 (0.82–0.97)
Portal Inflammation	0.81 (0.43–1.49)	0.49	0.81 (0.70–0.91)
Steatosis	1.35 (0.83–2.18)	0.22	0.77 (0.66–0.88)

Note: N = 109.

a Adjusted for treatment group, age < 13 or age >= 13, baseline BMI z-score, baseline outcome, and baseline HEI score.

b Area under the receiver operating characteristic curves for the adjusted model.

c Histological improvement is defined as a decrease in NAS to a score of 2 points or less and no worsening of fibrosis.

Abbreviations: AUROC, area under the receiver operating characteristic curve; BMI, body mass index; HEI, Healthy Eating Index; MASH, metabolic-associated steatohepatitis; NAS, nonalcoholic fatty liver disease activity score.

### Added sugar

When assessing the baseline relationships between added sugar and liver histologic features, there was a lower steatosis grade in those with < 10% added sugar (*p* = 0.03), but there were no differences in the fibrosis stage, NAS or the NAS components noted between added sugar ≤ 10% or > 10% (Table 5, Supplemental Table S2, http://links.lww.com/HC9/A690).

**TABLE 5:** Baseline relationship between sugar intake and liver histologic features^a^

	Total (N = 119)	Added sugar ≤ 10% (n = 68)	Added sugar > 10% (n = 51)	*p*-value^b^
NAS, mean (SD)	4.66 (1.39)	4.82 (1.42)	4.45 (1.32)	0.15
NAS ≥ 5, n (%)	67 (56.3)	39 (57.4)	28 (54.9)	0.79
Steatohepatitis diagnosis^b^	—	—	—	0.11
MAFLD	26 (21.8)	18 (26.5)	8 (15.7)	—
1a-borderline zone 3	16 (13.4)	5 (7.4)	11 (21.6)	—
1b-borderline zone 1	46 (38.7)	27 (39.7)	19 (37.3)	—
Definite	31 (26.1)	18 (26.5)	13 (25.5)	—
Fibrosis stage, mean (SD)	1.30 (1.05)	1.26 (1.06)	1.35 (1.06)	0.65
Fibrosis stage, n(%)				
0	29 (24.4)	19 (27.9)	10 (19.6)	0.15
1	49 (41.2)	24 (35.3)	25 (49.0)	—
2	17 (14.3)	13 (19.1)	4 (7.8)	—
3 or 4	24 (20.1)	12 (17.7)	12 (23.5)	—
Ballooning, mean (SD)	0.61 (0.76)	0.65 (0.77)	0.55 (0.76)	0.49
Ballooning, n(%)				
None	67 (56.3)	36 (52.9)	31 (60.8)	0.68
Few	32 (26.9)	20 (29.4)	12 (23.5)	—
Many	20 (16.8)	12 (17.6)	8 (15.7)	—
Lobular inflammation, mean (SD)	1.73 (0.71)	1.87 (0.73)	1.55 (0.64)	0.02
Lobular inflammation, n(%)				
1	50 (42)	23 (33.8)	27 (52.9)	0.051
2	51 (42.9)	31 (45.6)	20 (39.2)	—
3	18 (15.1)	14 (20.6)	4 (7.8)	—
Portal inflammation, mean (SD)	1.13 (0.51)	1.12 (0.47)	1.14 (0.57)	0.84
Portal inflammation, n(%)				
None	9 (7.6)	4 (5.9)	5 (9.8)	0.48
Mild	86 (72.3)	52 (76.5)	34 (66.7)	—
More than mild	24 (20.2)	12 (17.6)	12 (23.5)	—
Steatosis grade, mean (SD)	2.33 (0.77)	2.31 (0.72)	2.35 (0.84)	0.76
Steatosis grade, n(%)				
1	22 (18.5)	10 (14.7)	12 (23.5)	0.03
2	36 (30.3)	27 (39.7)	9 (17.6)	—
3	61 (51.3)	31 (45.6)	30 (58.8)	—

a NAS was assessed on a scale of 0-8, with higher scores showing more severe disease [the components of this measure are steatosis (assessed on a scale of 0-3), lobular inflammation (assessed on a scale of 0-3), and hepatocellular ballooning (assessed on a scale of 0-2)]. Fibrosis stage was assessed on a scale of 0-4 (by collapsing 1a, 1b, and 1c to 1), with higher scores showing more severe fibrosis.

b Independent samples *t*-tests for comparing means and chi-square test for comparing percentages.

Abbreviations: MAFLD, metabolic-associated fatty liver disease; NAS, nonalcoholic fatty liver disease activity score.

There were no associations between added sugar and changes in liver histologic features over time, including histologic improvement, NAS, its components, or fibrosis noted between added sugar ≤ 10% or > 10%. (Supplemental Table S3, http://links.lww.com/HC9/A690). However, like HEI, the adjusted model does predict improvement in several features, specifically histologic improvement, resolution of MASH, and improvement in NAS, fibrosis, lobular inflammation, and steatosis better than chance alone. There were no associations with changes in histologic features and a 1% difference in added sugar (Table 6).

**TABLE 6:** Changes in liver histologic features per 1% change in added sugar (N = 87)

	Change in score/ % increase in added sugar (95% CI)^a^	*p*-value	Adjusted change in score/ % increase in added sugar (95% CI)^a^	*p*
Change in score				
NAS	0.38 (−0.07 – 0.82)	0.09	0.13 (−0.25 – 0.52)	0.49
Fibrosis	−0.10 (−0.28 – 0.10)	0.31	−0.12 (−0.32 – 0.06)	0.20
Ballooning	0.08 (−1.20 – 0.44)	0.33	−0.05 (−0.20 – 0.11)	0.58
Lobular inflammation	0.17 (−0.01 – 0.35)	0.07	0.11 (−0.04 – 0.26)	0.16
Portal inflammation	0.003 (−0.13 – 0.14)	0.98	−0.02 (−0.17 – 0.13)	0.82
Steatosis	0.13 (−0.09 – 0.36)	0.25	0.07 (−0.14 – 0.29)	0.53

a Unit change in score for each additional added sugar % of calories increase from bootstrapped linear regression based on 1000 samples.

b Adjusted change in score/ % increase in added sugar (95% CI).

Abbreviation: NAS, nonalcoholic fatty liver disease activity score.

### Protein

When compared against protein intake as a percentage of total calories (≤ 20% or > 20%), there were no associations in fibrosis, NAS, or the NAS components at baseline (Supplemental Table S4, http://links.lww.com/HC9/A690, S5, http://links.lww.com/HC9/A690). There were no associations between baseline protein intake and changes in liver histologic features over time (Table 7), nor were there any associations with changes in liver histologic features with a 1% difference in protein intake (Supplemental Table S6, http://links.lww.com/HC9/A690).

**TABLE 7:** Association between 1% change in protein intake and changes in liver histologic features over time

	OR_ADJ_ (95% CI)/% increase in protein	*p*	AUROC^b^ (95% CI)
Histologic improvement^c^	1.02 (0.91–1.13)	0.74	0.69 (0.57–0.80)
Resolution of MASH	1.14 (0.99–1.31)	0.07	0.72 (0.57–0.86)
≥ 1 point improvement			
NAS	0.97 (0.87–1.09)	0.60	0.78 (0.69–0.88)
Fibrosis	0.98 (0.86–1.13)	0.78	0.80 (0.71–0.89)
Ballooning	1.10 (0.90–1.33)	0.35	0.94 (0.90–0.99)
Lobular inflammation	1.05 (0.90–1.21)	0.55	0.89 (0.82–0.96)
Portal inflammation	0.99 (0.86–1.13)	0.83	0.79 (0.67–0.91)
Steatosis	1.03 (0.98–1.08)	0.22	0.77 (0.66–0.88)

Note: N = 87.

a vORADJ (95% CI)/% increase in protein.

b Area under the receiver operating characteristic curves for the adjusted model.

c Histological improvement is defined as a decrease in NAS to a score of 2 points or less and no worsening of fibrosis.

Abbreviation: AUROC, area under the receiver operating characteristic curve; NAS, nonalcoholic fatty liver disease activity score.

### Fat

Higher fat (% of total calories) consumption was associated with a lower lobular inflammation score [OR (95% CI) = 0.95 (0.91 to 1.00), *p* = 0.04, Table 8] at baseline. No other histologic feature was associated with fat intake at baseline. A 1% change in fat intake was negatively associated with improvement in ballooning [OR (95% CI) = 0.84 (0.72 to 0.99), *p* = 0.03]. (Supplemental Table S7, http://links.lww.com/HC9/A690) was as well as an association with changes in ballooning score after adjusting for covariates [B (95% CI) = 0.03 (0.01 to 0.05), *p* = 0.003] (Supplemental Table S8, http://links.lww.com/HC9/A690).

**TABLE 8:** Relationship between baseline liver histologic features^a^ and fat intake as the percentage of calories (N = 119)

	Total n (%) (N = 119)	Fat % of Calories Mean (SD) (n = 119)	OR (95% CI)^b^	*p*
NAS^a^ ≥ 5	67 (56.3)	30.5 (8.0)	0.97 (0.93–1.02)	0.29
Steatohepatitis diagnosis	—	—	0.91 (0.91–1.04)	0.07
MAFLD	26 (21.8)	32.9 (7.3)	—	—
1a-borderline zone 3	16 (13.4)	32.8 (6.1)	—	—
1b-borderline zone 1	46 (38.7)	29.3 (6.7)	—	—
Definite	31 (26.1)	31.7 (8.7)	—	—
Fibrosis stage	—	—	1.02 (0.98–1.07)	0.30
0	29 (24.4)	32.3 (7.2)	—	—
1	17 (14.3)	28.9 (6.9)	—	—
2	23 (19.3)	31.9 (9.9)	—	—
3 or 4	24 (20.1)	34.1 (5.3)	—	—
Ballooning	—	—	1.01 (0.96–1.06)	0.68
None	67 (56.3)	30.9 (6.7)	—	—
Few	32 (26.9)	31.7 (8.2)	—	—
Many	20 (16.8)	31.3 (8.6)	—	—
Lobular inflammation	—	—	0.95 (0.91–1.00)	0.04
1	50 (42)	33.0 (6.0)	—	—
2	51 (42.9)	29.8 (7.7)	—	—
3	18 (15.1)	30.1 (9.3)	—	—
Portal inflammation	—	—	1.00 (0.94–1.05)	0.87
None	9 (7.6)	30.2 (5.4)	—	—
Mild	86 (72.3)	31.4 (7.6)	—	—
More than mild	24 (20.2)	30.6 (7.5)	—	—
Steatosis grade	—	—	0.99 (0.95–1.04)	0.87
1	22 (18.5)	29.7 (8.4)	—	—
2	36 (30.3)	32.4 (7.8)	—	—
3	61 (51.3)	31.0 (6.7)	—	—

a NAS was assessed on a scale of 0-8, with higher scores showing more severe disease (the components of this measure are steatosis [assessed on a scale of 0-3], lobular inflammation [assessed on a scale of 0-3], and hepatocellular ballooning [assessed on a scale of 0-2]). Fibrosis stage assessed on a scale of 0-4 (by collapsing 1a,1b,1c to 1), with higher scores showing more severe fibrosis.

b Logistic regression for 2 category outcomes, ordinal logistic regression for ordinal outcomes. Test of proportionality was assessed for ordinal logistic regression, with the probability of higher/more severe outcome modeled. OR for each additional % of total calorie intake in carbohydrates.

Abbreviations: MAFLD, metabolic-associated fatty liver disease; NAS, nonalcoholic fatty liver disease activity score.

### Carbohydrates

There were no significant relationships between the baseline histologic features and carbohydrate intake (% of total calories) (Supplemental Table S9, http://links.lww.com/HC9/A690). Longitudinally, there was no relationship with changes in defined improvement in any of the histologic features and carbohydrates at baseline or as a change in carbohydrate intake (Supplemental Table S10, http://links.lww.com/HC9/A690). However, there is a negative association between a 1% change in carbohydrate intake and changes in ballooning score after adjusting for covariates [B (95% CI) = −0.03 (−0.05 to −0.01), *p* = 0.004] (Supplemental Table S11, http://links.lww.com/HC9/A690).

### HEI and dietary component

All children in the study received standardized intensive nutrition intervention. To assess if such intensive nutritional counseling, reinforced at each visit, was effective in changing the dietary intake of the participants, we evaluated HEI and a change in dietary components from baseline to the end of the study. While no differences in HEI (*p* = 0.58), total calories (*p* = 1.00), the percentage of total calories from fat (*p* = 0.69), or from carbohydrates (*p*=0.19) were noted, the percentage of calories from proteins slightly increased from baseline to the end of the study, *p*=0.01 (Supplemental Table S12, http://links.lww.com/HC9/A690).

We also evaluated if there were any differences in HEI or the dietary components noted longitudinally in placebo versus treatment groups since this data was collected as part of an interventional RCT. As shown in Supplemental Table S13, http://links.lww.com/HC9/A690, no such differences were apparent.

## DISCUSSION

NAFLD is a major global pediatric health condition.^19–22^ Given the paucity of therapeutics, healthy diet and exercise remain the cornerstone strategies for intervention.^1,2,23–25^ Since diet composition can have significant variability, objective assessment of the role of dietary components in pediatric MAFLD remains poorly understood.^5,26^

Leveraging a well-characterized cohort of children with biopsy-proven MAFLD, as part of the National Institute of Diabetes and Digestive and Kidney Diseases NASH CRN, we assessed the impact of individual food components and diet quality by utilizing the HEI on hepatic steatosis, inflammation, fibrosis, liver injury chemistries, and serum lipids, controlling for age, sex, BMI, medications, and puberty stage.

We found a strong correlation between serum lipid profiles and HEI. Indeed, it is known that dyslipidemia is frequently associated with metabolic syndrome and MAFLD,^27–29^ and lipid screening in children with MAFLD is warranted.^30^ In fact, serum triglycerides are known to strongly associate with NAFLD among the various markers of hyperlipidemia.^31,32^ We noted a strong negative correlation between serum triglyceride levels and HEI, with levels being the lowest in the high (healthier) HEI group. Additionally, we noted serum HDL cholesterol was highest in the high HEI group, aligning with data that a high HDL cholesterol level confers protection against metabolic syndrome and MAFLD.^33,34^

Obesity is a known driver for MAFLD, and its safe and effective treatment remains a major challenge in clinical medicine.^35–39^ Supporting the role of a healthy diet in mediating change in body weight,^1,2,29^ there was a significant inverse correlation between HEI as a continuous variable and the BMI z-score. Indeed, a high HEI was associated with a significantly lower body weight, underscoring the critically important link between healthy diet and body mass.

The other important focus was the liver injury chemistry markers. Although we expected variance, we did not find any difference in serum aspartate aminotransferase, alanine aminotransferase, gamma-glutamyl transferase, HbA1c, fasting serum glucose, and fasting serum insulin between the HEI groups.

Furthermore, while we expected HEI to correlate with NAS, there was a poor correlation between HEI and NAS score at baseline or in NAS over time and baseline HEI. Additionally, when evaluated longitudinally, there were no associations between changes in fibrosis, NAS, or the NAS components and the baseline HEI groups. When assessing individual NAS components, a higher HEI by 10 U was associated with lower lobular inflammation scores. Of note, after adjusting for age, baseline BMI, and HEI score, there were no associations with a change in HEI by each 10 U; however, the model including HEI did predict outcomes much better than chance alone based on the AUROC and corresponding 95% CI for resolution of MASH, histologic improvement, and an improvement by 1 point or greater in NAS, fibrosis, lobular and portal inflammation, and steatosis. These histological results are nevertheless somewhat different from those reported in current literature. Despite all participants receiving lifestyle counseling, they could plausibly reflect local, regional, and national diet differences and diet fads since the study population was distributed throughout the US continent. However, addressing these may need further insight from future studies.

This study also assessed the association of the major food components, carbohydrates, proteins, and fat, with MAFLD histology. In those consuming ≤ 10% added sugar, we noted a lower steatosis grade. However, in contrast to current perceptions,^11,40^ we did not note any significant associations between changes in liver histologic features over time and added sugar nor with each 1% difference in added sugar. Additionally, both in our cross-sectional and longitudinal analysis, there was no relationship found between histologic features and calories from carbohydrates. These findings need further research and assessment.

Although it is generally believed that a high protein intake is beneficial for patients with MAFLD,^41,42^ we did not find a relationship between the percentage of calories from protein and longitudinal changes in liver histologic features. No associations were noted for changes in liver histologic features with a change in protein intake by each 1% of calories.

When assessing fat intake, there is evidence that higher fat intake is detrimental.^43,44^ In this study, a higher percent of calories from fat was associated with higher lobular inflammation, but we did not find an association between changes in NAS and changes in fat intake. Perhaps the lobular inflammation portends long-term liver injury related to fat intake.

Limitations of the study include that despite 3 days of daily food intake collection at baseline and at the 52-week follow-up, there could be significant diet differences in the interval period as well as variability in individual reporting across the groups. In addition, the number of children with longitudinal data was relatively small, and given the many comparisons, the power to detect small differences is low; however, these signals suggest further testing in larger cohorts of children may be warranted. Additionally, a large percentage of the study population was of Hispanic ethnicity, and the follow-up data was 52 weeks, which may possibly prevent the generalizability of these results to all children with MAFLD. These analyses do not provide the causal relationship between diet and histology but the associations between these two factors.

Strengths of the study include that this was a well-phenotyped cohort, with diet measured at multiple time points and liver biopsies close in time to the collection of dietary data. Rather than focusing on a single dietary component, we took a comprehensive view of diet, using multiple-day collections and the Healthy Eating Index.

In summary, this study in a well-characterized cohort of children enrolled in the NASH CRN CyNCh trial with biopsy-proven MAFLD and comprehensive dietary assessment through the NDSR addresses significant knowledge gaps and highlights the role of dietary components in regulating metabolic risk factors and histologic liver injury. Relevantly, a healthier diet represented by a high HEI was associated with lower body weight, higher serum HDL cholesterol, and lower serum triglyceride levels. We also noted the association of liver histology driven by the addition of sugar and fat intake. This analysis underscores the importance of critically evaluating the impact of diet in children with MAFLD, and HEI-based assessments could serve as a blueprint for future dietary intervention-focused studies.

## Supplementary Material
