# Supplementary material for: Nutrition assessment and MASH severity in children using the Healthy Eating Index
Source: Hepatol Commun. 2023 Dec 7;7(12):e0320. doi: 10.1097/HC9.0000000000000320 (PMC10984667; doi:10.1097/HC9.0000000000000320)

**SUPPLEMENTARY MATERIAL:**

**Appendix 1. List of NASH CRN participating Centers**

**Clinical Centers**

- Children’s Memorial Hospital, Chicago, IL (Peter Whitington, MD)
- Cincinnati Children’s Hospital Medical Center, Cincinnati, OH (Stavra A. Xanthakos, MD)
- Columbia University, New York, NY (Joel Lavine, MD, PhD)
- Emory University, Atlanta, GA (Saul Karpen, MD, PhD)
- Indiana University, Indianapolis, IN (Jean Molleston, MD)
- Saint Louis University, St. Louis, MO (Ajay Jain, MD)
- Texas Children’s Hospital, Houston, TX (Sarah Barlow, MD)
- University of California, San Diego, CA (Jeffrey Schwimmer, MD)
- University of California, San Francisco, CA (Philip Rosenthal, MD)
- University of Washington, Seattle, WA (Karen Murray, MD)

**Data Coordinating Center**

- Johns Hopkins University, Baltimore, MD (James Tonascia, PhD)

**Appendix 2. List of inclusion / exclusion criteria for the CyNCh trial**

### Inclusion criteria

Patients must satisfy all of the following criteria to be eligible for enrollment:

- Children age 8-17 years inclusive.
- Liver biopsy within 90 days of screening visit and not more than 120 days before randomization.
- Clinical history consistent with NAFLD.
- Definite NAFLD based upon liver histology.
- No evidence of any other liver disease by clinical history or histological evaluation
- A histological severity of NAFLD Activity Score (NAS) ≥ 4.
- Sexually active female participants of childbearing potential (i.e., not surgically sterile [defined as tubal ligation, hysterectomy, or bilateral oophorectomy) must agree to utilize the same two acceptable forms of contraception from screening through completion of the study and to complete a pregnancy test at each study visit. The acceptable forms of contraception for this study include hormonal contraceptives (oral, implant, transdermal patch, or injection) at a stable dose for at least 1 month prior to screening, and barrier (condom with spermicide, diaphragm with spermicide). Sexual activity will be ascertained at each study visit for post-menarchal females and if sexually active, subject must verify use of the same 2 acceptable forms of contraception.
- Participants must be able to swallow cysteamine bitartrate DR capsules.
- Written informed consent from parent or legal guardian.
- Written informed assent from the child

### Exclusion criteria

Exclusions will not be based upon gender, race, or ethnicity. Participants with a current history of the following conditions or any other health issues that make it unsafe for them to participate in the opinion of the investigators:

- Inflammatory bowel disease (if currently active) or prior resection of small intestine
- Heart disease (e.g., myocardial infarction, heart failure, unstable arrhythmias)
- Seizure disorders
- Active coagulopathy
- Gastrointestinal ulcers/bleeding
- Renal dysfunction with a creatinine clearance < 90 mL/min/m^2^
- History of active malignant disease requiring chemotherapy or radiation within the past 12 months prior to randomization
- History of significant alcohol intake (AUDIT questionnaire) or inability to quantify alcohol consumption
- Chronic use (defined as more than 2 consecutive weeks in the past year) of medications known to cause hepatic steatosis or steatohepatitis including:
  - systemic glucocorticoids
  - tetracycline
  - anabolic steroids
  - valproic acid
  - salicylates
  - tamoxifen,
- The use of other known hepatotoxins within 90 days of liver biopsy or within 120 days of randomization
- Initiation of medications with the intent to treat NAFLD/NASH in the time period following liver biopsy and prior to randomization
- History of total parenteral nutrition (TPN) use in the year prior to screening
- History of bariatric surgery or planning to undergo bariatric surgery during study duration
- Clinically significant depression (patients hospitalized for suicidal ideations or suicide attempts within the past 12 months)
- Any female who is nursing, planning a pregnancy, known or suspected to be pregnant, or who has a positive pregnancy screen
- Non-compensated liver disease with any one of the following hematologic, biochemical, and serological criteria on entry into protocol:
  - Hemoglobin < 10 g/dL
  - White blood cell (WBC) < 3,500 cells/mm3 of blood
  - Neutrophil count < 1,500 cells/mm3 of blood
  - Platelets < 130,000 cells/mm3 of blood
  - Direct bilirubin > 1.0 mg/dL
  - Total bilirubin >3 mg/dL
  - Albumin < 3.2 g/dL
  - International normalized ratio (INR) > 1.4
- Poorly controlled diabetes mellitus (hemoglobin A1c (HbA1c) > 9%)
- Evidence of other chronic liver disease:
  - Biopsy consistent with histological evidence of autoimmune hepatitis
  - Serum hepatitis B surface antigen (HBsAg) positive
  - Serum hepatitis C antibody (anti-HCV) positive
  - Iron/total iron binding capacity (TIBC) ratio (transferrin saturation) > 45% with histological evidence of iron overload
  - Alpha-1-antitrypsin (A1AT) phenotype/genotype ZZ or SZ
  - Wilson’s disease
- Children who are currently enrolled in a clinical trial or who have received an investigational study drug within 180 days of screening or liver biopsy
- Subjects who are not able or willing to comply with the protocol or have any other condition that would impede compliance or hinder completion of the study; in the opinion of the investigator
- Failure to give informed consent

**Appendix 3. Supplementary Tables**

**Supplementary Table T1: Changes in liver histologic features per 10-unit change in HEI**

|  | **Change in score/ 10-unit change in HEI (95% CI)^a^** | **p-value** | **Adjusted Change in score/ 10-unit change in HEI^b^ (95% CI) ^a^** | **p-value** |
| --- | --- | --- | --- | --- |
| **Change in Score** |  |  |  |  |
| NAS | -0.29 (-0.67 – 0.04) | 0.10 | -0.37 (-0.74 – 0.02) | 0.05 |
| Fibrosis | -0.07 (-0.24 – 0.09) | 0.40 | -0.11 (-0.30 – 0.07) | 0.23 |
| Ballooning | -0.07 (-0.19 – 0.05) | 0.28 | -0.07 (-0.20 – 0.06) | 0.27 |
| Lobular Inflammation | -0.12 (-0.25 – 0.01) | 0.08 | -0.18 (-0.30 - -0.07) | 0.007 |
| Portal Inflammation | -0.04 (-0.12 – 0.03) | 0.27 | -0.04 (-0.13 – 0.06) | 0.40 |
| Steatosis | -0.10 (-0.28 – 0.06) | 0.28 | -0.11 (-0.32 – 0.08) | 0.32 |

a) Unit change in score for each additional 10 unit increase of HEI score from bootstrapped linear regression based on 1000 samples.

b) Adjusted for treatment group, age < 13 or age >= 13, baseline BMI z-score, baseline outcome, and baseline HEI score.

**Supplementary Table T2: Relationship between baseline liver histologic features^a^ and sugar intake as percentage of calories (N = 119).**

|  | **Total**  **n (%)**  **(N = 119)** | **Added Sugar % Calories**  **Mean (SD)**  **(n = 119)** | **OR (95% CI)**^b^ | **p-value** |
| --- | --- | --- | --- | --- |
| NAS^a^ ≥ 5 | 67 (56.3) | 9.22 (5.28) | 0.91 (0.44 – 1.88) | 0.79 |
| Steatohepatitis Diagnosis |  |  | 1.05 (0.54 – 2.02) | 0.89 |
| MAFLD | 26 (21.8) | 9.46 (6.00) |  |  |
| 1a - borderline zone 3 | 16 (13.4) | 10.47 (3.92) |  |  |
| 1b - borderline zone 1 | 46 (38.7) | 8.79 (4.88) |  |  |
| Definite | 31 (26.1) | 10.07 (6.07) |  |  |
| Fibrosis stage |  |  | 1.16 (0.60 – 2.25) | 0.66 |
| 0 | 29 (24.4) | 9.30 (5.17) |  |  |
| 1 | 17 (14.3) | 10.05 (5.62) |  |  |
| 2 | 23 (19.3) | 7.07 (4.51) |  |  |
| 3 or 4 | 24 (20.1) | 10.33 (5.25) |  |  |
| Ballooning |  |  | 0.76 (0.37 – 1.54) | 0.44 |
| None | 67 (56.3) | 9.66 (5.18) |  |  |
| Few | 32 (26.9) | 9.10 (5.92) |  |  |
| Many | 20 (16.8) | 9.60 (5.07) |  |  |
| Lobular Inflammation |  |  | 0.42 (0.21 – 0.86) | 0.02 |
| 1 | 50 (42) | 10.82 (5.14) |  |  |
| 2 | 51 (42.9) | 8.85 (5.03) |  |  |
| 3 | 18 (15.1) | 7.64 (5.02) |  |  |
| Portal Inflammation |  |  | 1.12 (0.50 – 2.50) | 0.78 |
| None | 9 (7.6) | 11.06 (5.66) |  |  |
| Mild | 86 (72.3) | 9.24 (5.21) |  |  |
| More than mild | 24 (20.2) | 9.83 (5.75) |  |  |
| Steatosis Grade |  |  | 1.27 (0.64 – 2.55) | 0.49 |
| 1 | 22 (18.5) | 11.83 (6.78) |  |  |
| 2 | 36 (30.3) | 7.67 (4.24) |  |  |
| 3 | 61 (51.3) | 9.74 (5.03) |  |  |

a) NAFLD activity score (NAS) was assessed on a scale of 0-8, with higher scores showing more severe disease (the components of this measure are steatosis [assessed on a scale of 0-3], lobular inflammation [assessed on a scale of 0-3], and hepatocellular ballooning [assessed on a scale of 0-2]). Fibrosis stage assessed on a scale of 0-4 (by collapsing 1a,1b,1c to 1), with higher scores showing more severe fibrosis.

b) Logistic regression for 2 category outcomes, ordinal logistic regression for ordinal outcomes. Test of proportionality was assessed for ordinal logistic regression, with the probability of higher/more severe outcome modeled. OR for > 10 % added sugar vs ≤ 10 % added sugar.

**Supplementary Table T3: Association between changes in added sugar and liver histologic features over time. (N = 87)**

|  | **OR_ADJ_**  **(95% CI) / % increase in added sugar** | **p-value** | **AUROC^b^ (95% CI)** |
| --- | --- | --- | --- |
| Histologic improvement^c^ | 0.68 (0.39 – 1.21) | 0.19 | 0.65 (0.53 – 0.77) |
| Resolution of MASH | 0.72 (0.37 – 1.42) | 0.34 | 0.68 (0.53 – 0.82) |
| **≥ 1point improvement** |  |  |  |
| NAS | 0.86 (0.48 – 1.57) | 0.63 | 0.78 (0.69 – 0.88) |
| Fibrosis | 1.63 (0.86 – 3.09) | 0.14 | 0.83 (0.74 – 0.91) |
| Ballooning | 1.36 (0.50 – 3.71) | 0.55 | 0.95 (0.90 – 0.99) |
| Lobular inflammation | 0.71 (0.35 – 1.47) | 0.36 | 0.89 (0.82 – 0.96) |
| Portal inflammation | 1.50 (0.72 – 3.15) | 0.28 | 0.81 (0.69 – 0.93) |
| Steatosis | 0.72 (0.38 – 1.34) | 0.30 | 0.76 (0.66 – 0.86) |

a) Adjusted for treatment group, age < 13 or age >= 13, baseline BMI z-score, baseline added sugar intake (percent of total calories), and baseline outcome.

b) Area under the receiver operating characteristic curves for the adjusted model.

c) Histological improvement defined as a decrease in NAS to a score of 2 points or less and no worsening of fibrosis.

**Supplementary Table T4: Baseline relationship between protein intake and liver histologic features^a^**

|  | **Total**  **(N = 119)** | **Protein**  **≤ 20%**  **(n = 74)** | **Protein**  **> 20 %**  **(n = 45)** | **p-value^b^** |
| --- | --- | --- | --- | --- |
| NAS, mean (sd) | 4.66 (1.39) | 4.51 (1.36) | 4.91 (1.41) | 0.13 |
| NAS ≥ 5, n (%) | 67 (56.3) | 38 (51.4) | 29 (64.4) | 0.16 |
| Steatohepatitis Diagnosis^b^ |  |  |  | 0.98 |
| MAFLD | 26 (21.8) | 17 (23.0) | 9 (20.0) |  |
| 1a - borderline zone 3 | 16 (13.4) | 10 (13.5) | 6 (13.3) |  |
| 1b - borderline zone 1 | 46 (38.7) | 28 (37.8) | 18 (40.0) |  |
| Definite | 31 (26.1) | 19 (25.7) | 12 (26.7) |  |
| Fibrosis stage, mean (sd) | 1.30 (1.05) | 1.26 (1.07) | 1.40 (1.07) | 0.48 |
| Fibrosis stage, n (%) |  |  |  | 0.11 |
| 0 | 29 (24.4) | 19 (25.7) | 10 (22.2) |  |
| 1 | 49 (41.2) | 33 (44.6) | 16 (35.6) |  |
| 2 | 17 (14.3) | 6 (8.1) | 11 (24.4) |  |
| 3 or 4 | 24 (20.1) | 16 (21.6) | 8 (17.8) |  |
| Ballooning, mean (sd) | 0.61 (0.76) | 0.58 (0.74) | 0.64 (0.80) | 0.66 |
| Ballooning, n (%) |  |  |  |  |
| None | 67 (56.3) | 42 (56.8) | 25 (55.6) | 0.74 |
| Few | 32 (26.9) | 21 (28.4) | 11 (24.4) |  |
| Many | 20 (16.8) | 11 (14.9) | 9 (20.0) |  |
| Lobular Inflammation, mean (sd) | 1.73 (0.71) | 1.69 (0.70) | 1.80 (0.73) | 0.41 |
| Lobular Inflammation, n (%) |  |  |  |  |
| 1 | 50 (42) | 33 (44.6) | 17 (37.8) | 0.71 |
| 2 | 51 (42.9) | 31 (41.9) | 20 (44.4) |  |
| 3 | 18 (15.1) | 10 (13.5) | 8 (17.8) |  |
| Portal Inflammation, mean (sd) | 1.13 (0.51) | 1.15 (0.54) | 1.09 (0.47) | 0.54 |
| Portal Inflammation, n (%) |  |  |  |  |
| None | 9 (7.6) | 6 (8.1) | 3 (6.7) | 0.56 |
| Mild | 86 (72.3) | 51 (68.9) | 35 (77.8) |  |
| More than mild | 24 (20.2) | 17 (23.0) | 7 (15.6) |  |
| Steatosis Grade, mean (sd) | 2.33 (0.77) | 2.24 (0.81) | 2.47 (0.69) | 0.13 |
| Steatosis Grade, n (%) |  |  |  |  |
| 1 | 22 (18.5) | 17 (23.0) | 5 (11.1) | 0.25 |
| 2 | 36 (30.3) | 22 (29.7) | 14 (31.1) |  |
| 3 | 61 (51.3) | 35 (47.3) | 26 (57.8) |  |

a) NAFLD activity score (NAS) was assessed on a scale of 0-8, with higher scores showing more severe disease (the components of this measure are steatosis [assessed on a scale of 0-3], lobular inflammation [assessed on a scale of 0-3], and hepatocellular ballooning [assessed on a scale of 0-2]). Fibrosis stage assessed on a scale of 0-4 (by collapsing 1a,1b,1c to 1), with higher scores showing more severe fibrosis.

b) Independent samples t-tests for comparing means and Chi-square test for comparing percentages.

**Supplementary Table T5: Relationship between baseline liver histologic features^a^ and protein intake as percentage of calories (N = 119).**

|  | **Total**  **n (%)**  **(N = 119)** | **Protein % of Calories**  **Mean (SD)**  **(n = 119)** | **OR (95% CI)**^b^ | **p-value** |
| --- | --- | --- | --- | --- |
| NAS^a^ ≥ 5 | 67 (56.3) | 19.7 (5.3) | 1.72 (0.80 – 3.68) | 0.16 |
| Steatohepatitis Diagnosis |  |  | 1.12 (0.57 – 2.19) | 0.75 |
| MAFLD | 26 (21.8) | 17.7 (3.9) |  |  |
| 1a - borderline zone 3 | 16 (13.4) | 18.3 (3.9) |  |  |
| 1b - borderline zone 1 | 46 (38.7) | 19.9 (5.4) |  |  |
| Definite | 31 (26.1) | 19.1 (5.0) |  |  |
| Fibrosis stage |  |  | 1.30 (0.66 – 2.55) | 0.45 |
| 0 | 29 (24.4) | 18.1 (3.3) |  |  |
| 1 | 17 (14.3) | 18.8 (4.4) |  |  |
| 2 | 23 (19.3) | 22.9 (7.7) |  |  |
| 3 or 4 | 24 (20.1) | 17.6 (3.4) |  |  |
| Ballooning |  |  | 1.13 (0.55 – 2.31) | 0.74 |
| None | 67 (56.3) | 18.6 (4.6) |  |  |
| Few | 32 (26.9) | 19.4 (5.0) |  |  |
| Many | 20 (16.8) | 19.7 (5.5) |  |  |
| Lobular Inflammation |  |  | 1.34 (0.67 – 2.70) | 0.41 |
| 1 | 50 (42) | 17.9 (4.3) |  |  |
| 2 | 51 (42.9) | 19.5 (5.3) |  |  |
| 3 | 18 (15.1) | 20.5 (4.6) |  |  |
| Portal Inflammation |  |  | 0.76 (0.33 – 1.73) | 0.51 |
| None | 9 (7.6) | 18.3 (4.8) |  |  |
| Mild | 86 (72.3) | 19.4 (4.8) |  |  |
| More than mild | 24 (20.2) | 17.7 (5.2) |  |  |
| Steatosis Grade |  |  | 1.68 (0.82 – 3.45) | 0.16 |
| 1 | 22 (18.5) | 16.8 (4.2) |  |  |
| 2 | 36 (30.3) | 19.5 (4.3) |  |  |
| 3 | 61 (51.3) | 19.5 (5.2) |  |  |

a) NAFLD activity score (NAS) was assessed on a scale of 0-8, with higher scores showing more severe disease (the components of this measure are steatosis [assessed on a scale of 0-3], lobular inflammation [assessed on a scale of 0-3], and hepatocellular ballooning [assessed on a scale of 0-2]). Fibrosis stage assessed on a scale of 0-4 (by collapsing 1a,1b,1c to 1), with higher scores showing more severe fibrosis.

b) Logistic regression for 2 category outcomes, ordinal logistic regression for ordinal outcomes. Test of proportionality was assessed for ordinal logistic regression, with the probability of higher/more severe outcome modeled. OR for protein intake 20 % > of calories vs protein intake ≤ 20 % of calories.

**Supplementary Table T6: Changes in liver histologic features per 1% change in protein intake (N = 87)**

|  | **Change in score/**  **% increase in protein (95% CI)^a^** | **p-value** | **Adjusted Change in score/ % increase in protein (95% CI) ^a^** | **p-value** |
| --- | --- | --- | --- | --- |
| **Change in Score** |  |  |  |  |
| NAS | 0.003 (-0.07 – 0.09) | 0.95 | 0.007 (-0.07 – 0.09) | 0.89 |
| Fibrosis | 0.03 (-0.002 – 0.07) | 0.06 | -0.0003 (-0.04 – 0.04) | 0.98 |
| Ballooning | 0.01 (-0.02 – 0.04) | 0.36 | 0.001 (-0.03 – 0.03) | 0.95 |
| Lobular inflammation | 0.01 (-0.02 – 0.05) | 0.46 | 0.007 (-0.02 – 0.04) | 0.65 |
| Portal inflammation | 0.002 (-0.03 – 0.03) | 0.89 | 0.005 (-0.03 – 0.02) | 0.69 |
| Steatosis | -0.03 (-0.06 – 0.02) | 0.19 | -0.007 (-0.05 – 0.04) | 0.73 |

a) Unit change in score for each additional protein % of calories increase from bootstrapped linear regression based on 1000 samples.

b) Adjusted for treatment group, age < 13 or age >= 13, baseline BMI z-score, baseline outcome, and baseline protein intake (percent of total calories).

**Supplementary Table T7: Association between changes in fat intake and liver histologic features over time. (N = 87)**

|  | **OR_ADJ_**  **(95% CI) / % increase in fat** | **p-value** | **AUROC^b^ (95% CI)** |
| --- | --- | --- | --- |
| Histologic improvement^c^ | 0.93 (0.86 – 1.01) | 0.07 | 0.67 (0.55 – 0.79) |
| Resolution of MASH | 0.99 (0.90 – 1.10) | 0.88 | 0.68 (0.53 – 0.83) |
| **≥ 1point improvement** |  |  |  |
| NAS | 0.96 (0.89 – 1.04) | 0.29 | 0.79 (0.70 – 0.89) |
| Fibrosis | 1.01 (0.92 – 1.10) | 0.86 | 0.81 (0.72 – 0.90) |
| Ballooning | 0.84 (0.72 – 0.99) | 0.03 | 0.95 (0.92 – 0.99) |
| Lobular inflammation | 0.93 (0.85 – 1.03) | 0.16 | 0.89 (0.82 – 0.96) |
| Portal inflammation | 1.01 (0.92 – 1.12) | 0.81 | 0.82 (0.71 – 0.93) |
| Steatosis | 1.02 (0.93 – 1.10) | 0.72 | 0.75 (0.64 – 0.86) |

a) Adjusted for treatment group, age < 13 or age >= 13, baseline BMI z-score, baseline fat intake (percent of total calories), and baseline outcome.

b) Area under the receiver operating characteristic curves for the adjusted model.

c) Histological improvement defined as a decrease in NAS to a score of 2 points or less and no worsening of fibrosis.

**Supplementary Table T8: Changes in liver histologic features per 1% change in fat intake (N = 87)**

|  | **Change in score/**  **% increase in fat (95% CI)^a^** | **p-value** | **Adjusted Change in score/ % increase in fat (95% CI) ^a^** | **p-value** |
| --- | --- | --- | --- | --- |
| **Change in Score** |  |  |  |  |
| NAS | 0.02 (-0.04 – 0.08) | 0.46 | 0.05 (-0.001 – 0.10) | 0.04 |
| Fibrosis | -0.001 (-0.03 – 0.02) | 0.93 | -0.006 (-0.03 – 0.02) | 0.61 |
| Ballooning | 0.01 (-0.02 – 0.03) | 0.47 | 0.03 (0.01 – 0.05) | 0.003 |
| Lobular inflammation | 0.01 (-0.02 – 0.03) | 0.50 | 0.02 (-0.007 – 0.03) | 0.17 |
| Portal inflammation | 0.01 (-0.01 – 0.02) | 0.44 | 0.01 (-0.01 – 0.02) | 0.47 |
| Steatosis | 0.004 (-0.03 – 0.04) | 0.79 | 0.01 (-0.02 – 0.04) | 0.63 |

a) Unit change in score for each additional fat % of calories increase from bootstrapped linear regression based on 1000 samples.

b) Adjusted for treatment group, age < 13 or age >= 13, baseline BMI z-score, baseline outcome, and baseline fat intake (percent of total calories).

**Supplementary Table T9: Relationship between baseline liver histologic features^a^ and carbohydrate intake as percentage of calories (N = 119).**

|  | **Total**  **n (%)**  **(N = 119)** | **Carbohydrates % of Calories**  **Mean (SD)**  **(n = 119)** | **OR (95% CI)**^b^ | **p-value** |
| --- | --- | --- | --- | --- |
| NAS^a^ ≥ 5 | 67 (56.3) | 49.7 (7.8) | 0.99 (0.95 – 1.05) | 0.82 |
| Steatohepatitis Diagnosis |  |  | 1.02 (0.97 – 1.07) | 0.55 |
| MAFLD | 26 (21.8) | 49.5 (8.6) |  |  |
| 1a - borderline zone 3 | 16 (13.4) | 48.9 (6.4) |  |  |
| 1b - borderline zone 1 | 46 (38.7) | 50.8 (6.7) |  |  |
| Definite | 31 (26.1) | 49.1 (7.9) |  |  |
| Fibrosis stage |  |  | 0.96 (0.92 – 1.01) | 0.10 |
| 0 | 29 (24.4) | 49.7 (8.2) |  |  |
| 1 | 17 (14.3) | 52.3 (7.1) |  |  |
| 2 | 23 (19.3) | 45.2 (6.6) |  |  |
| 3 or 4 | 24 (20.1) | 48.3 (5.4) |  |  |
| Ballooning |  |  | 0.97 (0.93 – 1.02) | 0.27 |
| None | 67 (56.3) | 50.6 (7.0) |  |  |
| Few | 32 (26.9) | 48.8 (8.3) |  |  |
| Many | 20 (16.8) | 49.0 (6.9) |  |  |
| Lobular Inflammation |  |  | 1.02 (0.97 – 1.06) | 0.53 |
| 1 | 50 (42) | 49.1 (6.2) |  |  |
| 2 | 51 (42.9) | 50.7 (7.9) |  |  |
| 3 | 18 (15.1) | 49.4 (8.8) |  |  |
| Portal Inflammation |  |  | 1.02 (0.97 – 1.08) | 0.41 |
| None | 9 (7.6) | 51.4 (5.3) |  |  |
| Mild | 86 (72.3) | 49.1 (7.2) |  |  |
| More than mild | 24 (20.2) | 51.7 (8.3) |  |  |
| Steatosis Grade |  |  | 0.97 (0.92 – 1.01) | 0.17 |
| 1 | 22 (18.5) | 53.5 (8.4) |  |  |
| 2 | 36 (30.3) | 48.1 (7.2) |  |  |
| 3 | 61 (51.3) | 49.5 (6.7) |  |  |

a) NAFLD activity score (NAS) was assessed on a scale of 0-8, with higher scores showing more severe disease (the components of this measure are steatosis [assessed on a scale of 0-3], lobular inflammation [assessed on a scale of 0-3], and hepatocellular ballooning [assessed on a scale of 0-2]). Fibrosis stage assessed on a scale of 0-4 (by collapsing 1a,1b,1c to 1), with higher scores showing more severe fibrosis.

b) Logistic regression for 2 category outcomes, ordinal logistic regression for ordinal outcomes. Test of proportionality was assessed for ordinal logistic regression, with the probability of higher/more severe outcome modeled. OR for each additional % of total calorie intake in carbohydrates.

**Supplementary Table T10: Association between carbohydrate intake and changes in liver histologic features over time. (N = 87)**

|  | **OR_ADJ_**  **(95% CI) / % increase in carbohydrates** | **p-value** | **AUROC^b^ (95% CI)** |
| --- | --- | --- | --- |
| Histologic improvement^c^ | 1.06 (0.99 – 1.34) | 0.11 | 0.65 (0.53 – 0.78) |
| Resolution of MASH | 0.96 (0.87 – 1.05) | 0.34 | 0.68 (0.52 – 0.83) |
| **≥ 1point improvement** |  |  |  |
| NAS | 1.05 (0.98 – 1.14) | 0.18 | 0.80 (0.71 – 0.89) |
| Fibrosis | 1.00 (0.92 – 1.09) | 0.97 | 0.83 (0.74 – 0.91) |
| Ballooning | 1.11 (0.98 – 1.26) | 0.09 | 0.95 (0.90 – 0.99) |
| Lobular inflammation | 1.05 (0.96 – 1.15) | 0.31 | 0.89 (0.82 – 0.96) |
| Portal inflammation | 1.00 (0.92 – 1.10) | 0.92 | 0.80 (0.67 – 0.92) |
| Steatosis | 1.00 (0.92 – 1.08) | 0.94 | 0.75 (0.65 – 0.86) |

a) Adjusted for treatment group, age < 13 or age >= 13, baseline BMI z-score, baseline carbohydrate intake (percent of total calories), and baseline outcome.

b) Area under the receiver operating characteristic curves for the adjusted model.

c) Histological improvement defined as a decrease in NAS to a score of 2 points or less and no worsening of fibrosis.

**Supplementary Table T11: Changes in liver histologic features per 1% change in carbohydrate intake. (N = 87)**

|  | **Change in score/**  **% increase in carbohydrates (95% CI)^a^** | **p-value** | **Adjusted Change in score/ % increase in carbohydrates (95% CI) ^a^** | **p-value** |
| --- | --- | --- | --- | --- |
| **Change in Score** |  |  |  |  |
| NAS | -0.02 (-0.08 – 0.04) | 0.54 | -0.05 (-0.10 – 0.002) | 0.06 |
| Fibrosis | -0.01 (-0.03 – 0.01) | 0.27 | -0.005 (-0.02 – 0.03) | 0.69 |
| Ballooning | -0.01 (-0.03 – 0.01) | 0.23 | -0.03 (-0.05 – -0.01) | 0.004 |
| Lobular inflammation | -0.01 (-0.04 – 0.01) | 0.31 | -0.02 (-0.04 – 0.003) | 0.12 |
| Portal inflammation | -0.01 (-0.02 – 0.01) | 0.50 | -0.004 (-0.02 – 0.01) | 0.65 |
| Steatosis | 0.01 (-0.02 – 0.03) | 0.62 | -0.002 (-0.03 – 0.02) | 0.88 |

a) Unit change in score for each additional carbohydrate % of calories increase from bootstrapped linear regression based on 1000 samples.

b) Adjusted for treatment group, age < 13 or age >= 13, baseline BMI z-score, baseline outcome, and baseline carbohydrate intake (percent of total calories).

**Supplementary Table T12: Change in HEI and dietary components from baseline to the end of study.**

|  | **Baseline**  **(n = 119)**  **mean (sd)** | **Follow-up**  **(n = 87)**  **mean (sd)** | **Change**  **(n = 87)**  **mean (sd)** | **p-value** |
| --- | --- | --- | --- | --- |
| HEI | 53.7 (12.3) | 53.2 (12.5) | -0.75 (12.6) | 0.58 |
| Total calories (kcal) | 1471.8 (430.0) | 1524.4 (498.3) | 0.01 (481.1) | 1.00 |
| Calories from fat (%) | 31.2 (7.4) | 31.3 (6.9) | -0.3 (7.5) | 0.69 |
| Calories from Carbohydrates (%) | 49.8 (7.4) | 48.7 (7.3) | -1.2 (8.3) | 0.19 |
| Calories from Protein (%) | 19.0 (4.9) | 20.0 (5.6) | 1.5 (5.3) | 0.01 |

**Supplementary Table T13: Change from baseline in dietary components. (N = 87)**

|  | **placebo**  **mean (sd)** | **treatment**  **mean (sd)** | **p-value** |
| --- | --- | --- | --- |
| HEI | 0.57 (12.3) | -1.99 (12.9) | 0.35 |
| Total calories (kcal) | 0.28 (446.1) | -0.23 (516.7) | 1.00 |
| Calories from fat (%) | -0.84 (6.5) | 0.15 (8.4) | 0.54 |
| Calories from Carbohydrates (%) | -1.17 (8.2) | -1.21 (8.5) | 0.98 |
| Calories from Protein (%) | 2.01 (5.6) | 1.1 (4.9) | 0.41 |

**Supplementary Figure S1: Change in NAS and HEI**


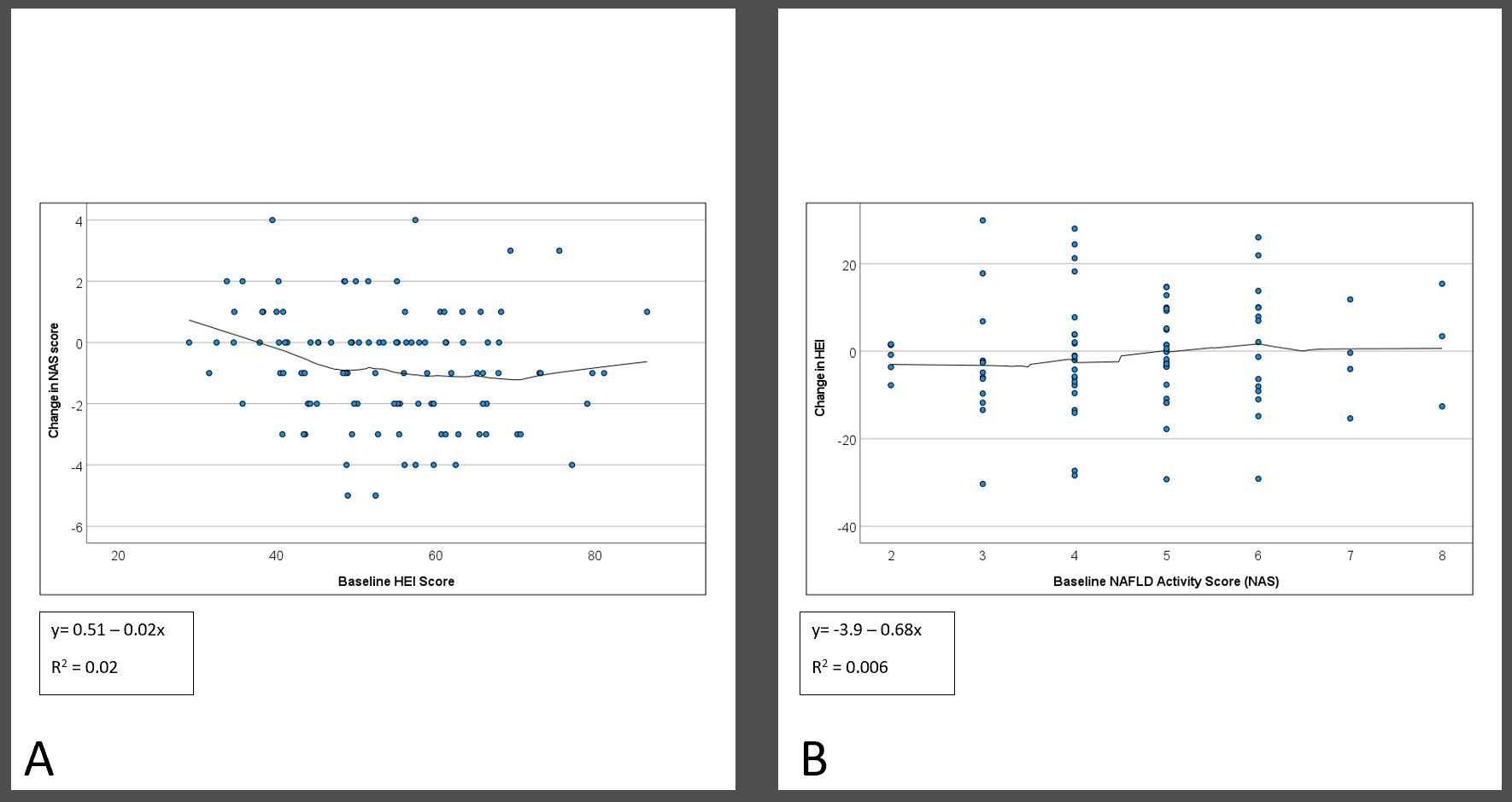

Supplement: Supplementary file 1 [file hc9-7-e0320-s001.docx]
